# Field evaluation of the diagnostic performance of EasyScan GO: a digital malaria microscopy device based on machine-learning

**DOI:** 10.1186/s12936-022-04146-1

**Published:** 2022-04-12

**Authors:** Debashish Das, Ranitha Vongpromek, Thanawat Assawariyathipat, Ketsanee Srinamon, Kalynn Kennon, Kasia Stepniewska, Aniruddha Ghose, Abdullah Abu Sayeed, M. Abul Faiz, Rebeca Linhares Abreu Netto, Andre Siqueira, Serge R. Yerbanga, Jean Bosco Ouédraogo, James J. Callery, Thomas J. Peto, Rupam Tripura, Felix Koukouikila-Koussounda, Francine Ntoumi, John Michael Ong’echa, Bernhards Ogutu, Prakash Ghimire, Jutta Marfurt, Benedikt Ley, Amadou Seck, Magatte Ndiaye, Bhavani Moodley, Lisa Ming Sun, Laypaw Archasuksan, Stephane Proux, Sam L. Nsobya, Philip J. Rosenthal, Matthew P. Horning, Shawn K. McGuire, Courosh Mehanian, Stephen Burkot, Charles B. Delahunt, Christine Bachman, Ric N. Price, Arjen M. Dondorp, François Chappuis, Philippe J. Guérin, Mehul Dhorda

**Affiliations:** 1 Infectious Diseases Data Observatory (IDDO), Oxford, UK; 2 WorldWide Antimalarial Resistance Network (WWARN), Oxford, UK; 3 Centre for Tropical Medicine and Global Health, Nuffield Department of Medicine, University of Oxford, Oxford, UK; 4 Institute of Global Health, University of Geneva, Geneva, Switzerland; 5 Faculty of Tropical Medicine, Mahidol-Oxford Tropical Medicine Research Unit, Mahidol University, Bangkok, Thailand; 6 Chittagong Medical College (CMC), Chattogram, Bangladesh; 7 Dev Care Foundation, Dhaka, Bangladesh; 8 Fundação de Medicina Tropical Dr Heitor Vieira Dourado, Manaus, Amazonas Brazil; 9 Oswaldo Cruz Foundation (Fiocruz), Rio de Janeiro, Brazil; 10 Institut Des Sciences Et Techniques (INSTech), Bobo-Dioulasso, Burkina Faso; 11 Fondation Congolaise Pour La Recherche Médicale (FCRM), Brazzaville, Congo; 12 Kenya Medical Research Institute (KEMRI), Nairobi, Kenya; 13 Tribhuvan University, Kathmandu, Nepal; 14 Global and Tropical Health Division, Menzies School of Health Research, Charles Darwin University, Darwin, NT Australia; 15 Faculty of Medicine, University Cheikh Anta Diop (UCAD), Dakar, Senegal; 16 Parasitology Reference Laboratory, National Institute for Communicable Diseases, Division of the National Health Laboratory Service, Johannesburg, South Africa; 17 Shoklo Malaria Research Unit, Mahidol-Oxford Tropical Medicine Research Unit, Faculty of Tropical Medicine, Mahidol University, Mae Sot, Thailand; 18 Department of Pathology, College of Health Science, Makerere University, Kampala, Uganda; 19 Infectious Diseases Research Collaboration (IDRC), Kampala, Uganda; 20 University of California, San Francisco, CA USA; 21 Global Health Labs, Bellevue, WA USA; 22 University of Oregon, Eugene, OR USA; 23 Division of Tropical and Humanitarian Medicine, Geneva University Hospitals and University of Geneva, Geneva, Switzerland

**Keywords:** Malaria, Light microscopy, Digital microscopy, Artificial intelligence, Diagnostic accuracy, Machine-learning

## Abstract

**Background:**

Microscopic examination of Giemsa-stained blood films remains the reference standard for malaria parasite detection and quantification, but is undermined by difficulties in ensuring high-quality manual reading and inter-reader reliability. Automated parasite detection and quantification may address this issue.

**Methods:**

A multi-centre, observational study was conducted during 2018 and 2019 at 11 sites to assess the performance of the EasyScan Go, a microscopy device employing machine-learning-based image analysis. Sensitivity, specificity, accuracy of species detection and parasite density estimation were assessed with expert microscopy as the reference. Intra- and inter-device reliability of the device was also evaluated by comparing results from repeat reads on the same and two different devices. This study has been reported in accordance with the Standards for Reporting Diagnostic accuracy studies (STARD) checklist.

**Results:**

In total, 2250 Giemsa-stained blood films were prepared and read independently by expert microscopists and the EasyScan Go device. The diagnostic sensitivity of EasyScan Go was 91.1% (95% CI 88.9–92.7), and specificity 75.6% (95% CI 73.1–78.0). With good quality slides sensitivity was similar (89.1%, 95%CI 86.2–91.5), but specificity increased to 85.1% (95%CI 82.6–87.4). Sensitivity increased with parasitaemia rising from 57% at < 200 parasite/µL, to ≥ 90% at > 200–200,000 parasite/µL. Species were identified accurately in 93% of *Plasmodium falciparum* samples (kappa = 0.76, 95% CI 0.69–0.83), and in 92% of *Plasmodium vivax* samples (kappa = 0.73, 95% CI 0.66–0.80). Parasite density estimates by the EasyScan Go were within ± 25% of the microscopic reference counts in 23% of slides.

**Conclusions:**

The performance of the EasyScan Go in parasite detection and species identification accuracy fulfil WHO-TDR Research Malaria Microscopy competence level 2 criteria. In terms of parasite quantification and false positive rate, it meets the level 4 WHO-TDR Research Malaria Microscopy criteria. All performance parameters were significantly affected by slide quality. Further software improvement is required to improve sensitivity at low parasitaemia and parasite density estimations.

*Trial registration* ClinicalTrials.gov number NCT03512678.

**Supplementary Information:**

The online version contains supplementary material available at 10.1186/s12936-022-04146-1.

## Background

Microscopic examination of Giemsa-stained blood films remains the primary reference method for the detection, identification and quantification of malaria parasitaemia. It continues to be routinely performed for laboratory confirmation of malaria infection in some countries, but is increasingly being replaced by rapid diagnostic tests (RDTs), which are much easier to use and do not require equipment or extensive training [1]. RDTs, however, are less sensitive for low-density infections, can only provide qualitative results, mostly only differentiate *Plasmodium falciparum* from all other plasmodial species, and are challenged by parasite lineages with deletions in genes coding for antigens detected by RDTs. The information on parasite density and species is required for clinical management of patients and is particularly important in the context of research studies on malaria prevention, diagnostics and treatment [2]. Thus microscopy remains a gold standard for clinical diagnosis and will continue to be used in malaria clinical research settings for the foreseeable future.

The validity and reliability of microscopy depend heavily on the availability and competence of laboratory technicians. Standardization of malaria microscopy is an ongoing challenge due to difficulties in attaining and maintaining high-quality manual reading and inter-reader consistency [3–6]. Automated systems combining hardware that capture images from a blood slide with malaria detection algorithms for analysis of the images to deliver parasite detection, species identification, and quantitation without user input may address some shortfalls of manual microscopy. Most research into algorithms has (i) focused on thin films, which are unsuitable for diagnosis and quantitation of lower-parasitaemia samples [7]; (ii) used small datasets without field validations [8]; and (iii) does not address the hardware step. However, some progress has been made on Giemsa-stained thick film algorithms [9–13] which are central to clinical use-cases. These algorithms apply convolutional neural networks (CNNs), a machine learning method that has been highly successful in image-related tasks [14, 15]. Such methods hold great potential for automated recognition of malaria parasites from standard Giemsa-stained blood films. The current study assesses such an automated device and algorithms.

A brief history of the system and its previous field trials, as reported in [10, 11, 16–19] is given here. A digital malaria microscopy device, with both hardware and software developed by Intellectual Ventures’ Global Good Fund, and operating on thick films only, was first tested in field settings in Thailand in 2014–2015 [10]. A second version of the thick film algorithm targeted both *Plasmodium falciparum* and *Plasmodium vivax* (as well as *Plasmodium ovale* and *Plasmodium malariae*), though only the *P. falciparum* branch is described [11]. In a test at the same site in Thailand plus an additional site in Indonesia in 2016–2017 [18], this version of the thick film algorithm had (compared to expert microscopy) a diagnostic sensitivity of 89%, a specificity of 97%, species identification (falciparum vs vivax) accuracy of 84%, and quantitation accuracy such that 30% of the parasite density estimates were within ± 25% of that derived by microscopic quantification. This thick film algorithm was also tested in a field trial in Peru [16], where it performed slightly worse than expert microscopy: using PCR as a reference, diagnostic sensitivity was 72% (vs 68% for microscopy), specificity was 85% (vs 100% for microscopy), limit of detection was roughly 100–150 p/uL, and species identification accuracy was 90% on positive samples. An improved iteration of the software, with an added thin film module (described in [17]), was combined with the EasyScan GO, a production prototype device built by Motic using software also licensed to Motic, and tested on a standard WHO slide set [19]. It achieved WHO level 1 diagnostic (implying a limit of detection of roughly 100 p/uL), level 1 quantitation accuracy, and level 2 species identification performance. The same setup was also tested in an extended trial at London’s Hospital for Tropical Diseases (manuscript in preparation). The EasyScan GO plus the thick film algorithm (no thin film module) was deployed in the study reported here, which evaluated the system in diverse transmission and epidemiological malaria contexts (11 sites in 11 countries).

The specific objectives of the study were to assess the diagnostic performance of the production prototype of the EasyScan Go with respect to parasite detection, species identification, and parasite density estimation using expert microscopy as a reference standard.

## Methods

### Study site, design, and oversight

**Fig. 1:**
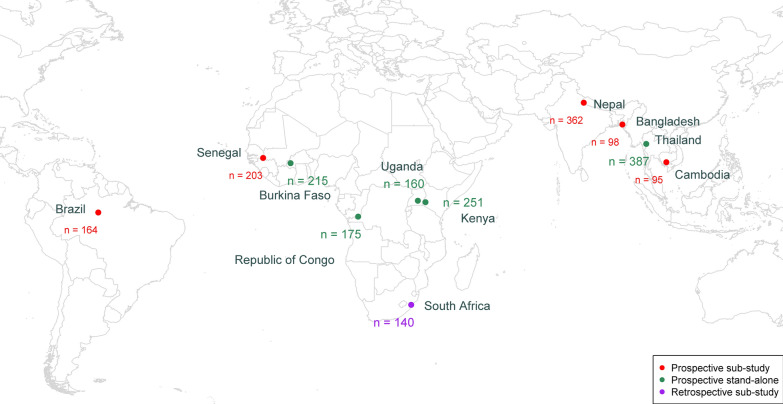
Study sites (11 sites, n = 2250 slides)

A multi-centre, observational study was conducted during 2018 and 2019 at 11 sites in 11 countries: Burkina Faso, Kenya, Republic of the Congo, Senegal, South Africa, Uganda in Africa, Bangladesh, Cambodia, Nepal, Thailand in Asia, and Brazil in South America (Fig. 1). The evaluation of the EasyScan Go device was conducted as a ‘sub-study’ alongside ongoing studies of malaria treatments or diagnostics in 6 sites (Bangladesh, Brazil, Cambodia, Nepal, Senegal, and South Africa), or as a ‘stand-alone’ study in 5 sites (Burkina Faso, Kenya, Republic of the Congo, Thailand, and Uganda). Most studies used a cross-sectional design, except in Cambodia and South Africa, where slides were collected at inclusion and during follow-up visits. Further, the study was conducted as a prospective evaluation except for South Africa, where slides were read and data collection was performed retrospectively. The study has been reported in accordance with the Standards for Reporting Diagnostic accuracy studies (STARD) checklist (Additional file 1) [20].

### Ethical approval

The study was conducted in accordance with the International Conference on Harmonization Guidelines for Good Clinical Practice (ICH-GCP), applicable regulatory requirements, and the Declaration of Helsinki. Approval for the multi-centre study was granted by the Oxford Tropical Research Ethics Committee (Ref 514-18) and the national ethics committee and/or the institutional review board for each site. The multi-centre study was registered at ClinicalTrials.gov and assigned identifier NCT03512678. All study participants provided written informed consent.

### Study participants and samples

Participants were enrolled using convenience sampling with the following inclusion criteria: (i) age between 6 months and 75 years; (ii) history of fever in the past 48 h or measured temperature ≥ 37.5 °C; (iii) informed written consent or assent. Informed consent was obtained from parents of children under 18 years. Patients with signs of severe malaria at screening, as defined by the WHO, were excluded from the study [2]. A blood sample was obtained by finger prick or venepuncture to prepare Giemsa-stained blood films for microscopy and to perform RDT. After blood sampling, any patient with a diagnosis of malaria by microscopy or RDT received standard anti-malarial treatment in accordance with the national guidelines (stand-alone studies) or a treatment per the main trial protocol (sub-studies).

### Examination of blood films by light microscopy

Microscopy procedures, including slide preparation, staining, reading, and quality control, were performed according to the methods described in the WHO-TDR Research Malaria Microscopy Guidelines [21]. A micropipette was used to place 6 µL of blood for making a 12 mm diameter thick film using a slide template and 2–3 µL of blood for thin film. Both thick and thin films were stained with freshly prepared 3% v/v Giemsa solution in buffered water, pH 7.2 for 40–50 min, and air-dried completely. Asexual parasite density was estimated using the ‘per High Power Field’ (HPF) method or by counting against 500 WBC if actual WBC count from a haematology analyser was available (Additional file 2). In cases with more than 200 parasites in 10 HPF, parasite density was estimated by counting 2000 RBCs on the thin film. A slide was considered negative if no asexual parasites were found after examining 200 HPF on the thick smear. Gametocytes were not assessed. Each slide was read by two microscopists (a different pair at each site).

### Examination of blood films by the EasyScan Go

The EasyScan Go devices were donated by the Intellectual Ventures' Global Good Fund. Device set-up, calibration and training on the operation were performed according to the accompanying manuals at the study initiation training at each site. The same slide was read by expert microscopists and on the EasyScan Go device. All slides prepared for manual microscopy were first read on the thick film using the EasyScan Go device, and a thin film read was performed only when the parasite count on thick film by the EasyScan device was over 10,000 per µL. The first reading was usually performed immediately after the manual microscopy was done. A second read with EasyScan Go was also performed either immediately after the first read or after the recruitment of all participants.

#### Scanning

A comprehensive description of the imaging methods and the accompanying software developed by Intellectual Ventures’ Global Good Fund are found in [19] and an image of the EasyScan GO is included in the Additional file 3: Fig. S1. Briefly, slides were prepared for imaging by placing a drop of immersion oil and a coverslip on the blood film. The slide was then slotted into a holder before inserting it into the EasyScan Go device. The EasyScan GO has a 40x, 0.75 NA objective, and captures a stack of colour images (2048 × 1536 pixels) with 8.3 pixels/um pixel pitch and 0.5 um vertical spacing. The image stacks are necessary to ensure in-focus thumbnails of objects at different depths in the blood film. A total of 144 fields of view (stacks) were captured, containing 1000 to 6000 WBCs (~ 0.125 to 0.75 uL of blood). Capturing at least 0.1 uL of blood ensures a high likelihood that the imaged blood contains at least one or two parasites given a 50 p/uL parasitaemia infection, based on Poisson statistics (as reported in Supplementary material of [17]).

#### Algorithms

Full details of the malaria algorithm architecture and logical flow are found in [19]. Specific details of the thick film algorithm module are found in [11]. Thin film algorithms (not part of this study) are described in [17]. Briefly, the thick film algorithm automatically analyses the image stacks to detect and quantify parasites, and to identify species (i.e. falciparum vs vivax as a default for non-falciparum). The module first detects candidate objects by fast methods; then culls most of these objects with a fast (non-CNN) classifier; then applies a CNN model to the remaining candidates to classify them as parasite or distractor. Positive diagnosis depends on whether the count of suspected parasites (per WBC) exceeds a pre-set noise threshold. The algorithm also rates slide quality based on statistical characteristics of distractor objects, and raises a flag if poor quality is suspected.

The absence of a thin film module affects species identification and quantitation. While the thick film module readily distinguishes *Plasmodium falciparum* from non-falciparum infections, it does not distinguish between non-falciparum species and instead defaults all non-falciparum reads to “vivax”. The algorithm’s thick film quantitation accuracy is consistent up to roughly 100,000 p/uL, contrary to manual microscopy which switches to thin films at 16,000 p/uL [21]. Below 100,000 p/uL the much larger quantity of blood examined on thick film vs. thin film reduces the Poisson variability of the true parasite count [as reported in Supplementary material [17]]. However, above 100,000 p/uL the automated thick film reads become unreliable due to crowding.

#### Output report

Upon completion of the image scanning and analysis, an automatically-generated report indicates the diagnosis (presence/absence of malaria parasites), WBC count, and the species and parasite density if the slide diagnosis is positive. The report also displays a mosaic of suspected parasite thumbnails, and clicking on a thumbnail brings up the relevant field-of-view to allow easy examination by a clinician. A screenshot of a typical report, with a selected field-of-view, is shown in Additional file 4: Fig. S2. The acquisition and processing time for the thick film was 20–30 min. In this study, the scanned images and the results generated from the device were stored in external hard drives provided with the EasyScan Go device.

#### Algorithm versions and training data

During the study, an earlier algorithm version (1.11.6) was run, but it can be viewed as a placeholder. At the conclusion of the study, the saved image sets were re-analysed in the field with an updated version (1.15.17b, also used in [19]). For both versions, all diagnostic thresholds were pre-specified during algorithm development and targeted an operating point that would give specificity of 90% on validation sets from sites that provided training slides [19]. No samples from the sites tested in this study, and no interim findings or results, were used during any part of algorithm training or tuning, for either algorithm version. Thus the results for algorithm version 1.15.17b, reported here, represent true hold-out performance at new sites in new countries.

### Microscopy quality control measures

All sites used a standardized laboratory manual and standard operating procedures (SOPs) for malaria microscopy. Blood slide reading was performed independently by two qualified microscopists, who were blinded to individual microscopy results. The Obare Method Calculator (https://www.wwarn.org/obare-method-calculator) was used to identify discrepancies in the microscopy results requiring tie-breaker reads and to determine the consensus result [22]. The Obare Method Calculator is a Microsoft Excel-based tool to facilitate adherence to the recommendations for internal quality control (IQC) as per the Research Microscopy Method Manual, WHO 2015. The tool helps researchers to make systematic assessments of whether the results of two blood film reads are concordant or require a third read and reports consensus results. Blinded External Quality Control (EQC) of blood slides was performed by an expert microscopist with certified WHO Competence Level 1 at the WWARN Asia–Pacific Regional Centre in Bangkok, Thailand. A random selection of slides (either 20% or 25 positive and 25 negative slides per site, whichever was greater) were assessed for slide quality and diagnostic performance. Sites were classified as “first tier sites” vs. “second tier sites” by slide quality assessed with respect to the presence or absence of artefacts, staining quality, and smear quality (thickness, size). Standard indicators were calculated to assess diagnostic performance [21]. The study sites were provided with the EQC report and feedback on slide quality.

### Statistical considerations

At each site, a minimum of 80 malaria slides confirmed by light microscopy and a minimum of 80 malaria-negative slides were assessed. The sample size was calculated based upon an assumed diagnostic sensitivity and specificity of 95%, to ensure a 10% accuracy (i.e. the width of the estimated 95% confidence interval < 10%), assuming that up to 10% of subjects would be excluded due to missing or incomplete data [23]. This sample size allowed estimation of the binary kappa statistics with an accuracy of 0.07 for kappa values between 0.5 and 0.94. The main study outcomes were diagnostic performance indicators—sensitivity and specificity for malaria parasite detection, kappa statistics for parasite species identification [24], and Bland–Altman plots for parasite density estimation [25]. Intra- and inter-device reliability was also assessed by comparing results obtained by performing repeat reads on the same device at each site or a second EasyScan Go device. Study data were collected and managed using the Research Electronic Data Capture (REDCap) tools hosted at the University of Oxford [26, 27]. All analyses were conducted using Stata software, version 15.1 (StataCorp College Station, Texas, USA).

## Results

In total, 2,250 slides were evaluated, of which 969 (43.1%) tested positive by light microscopy (Additional file 5: Table S1). A majority of the slides were collected from male participants (53.9%, 1213/2250) with a mean (sd) age of 22.7 (17.4) years. Among microscopy positive slides, 624 (64.4%) were infected with *P. falciparum*, 327 (33.7%) with *P. vivax,* 8 (0.8%) with mixed *P. falciparum* and *P. vivax* infections and the remaining 10 (1.0%) with other mixed or non *P. falciparum*/*P. vivax* infections. The results presented here were obtained using the most recent version of the algorithm available at the time the analyses were performed (version1.15.17b).

### Parasite detection by the EasyScan Go

**Fig. 2:**
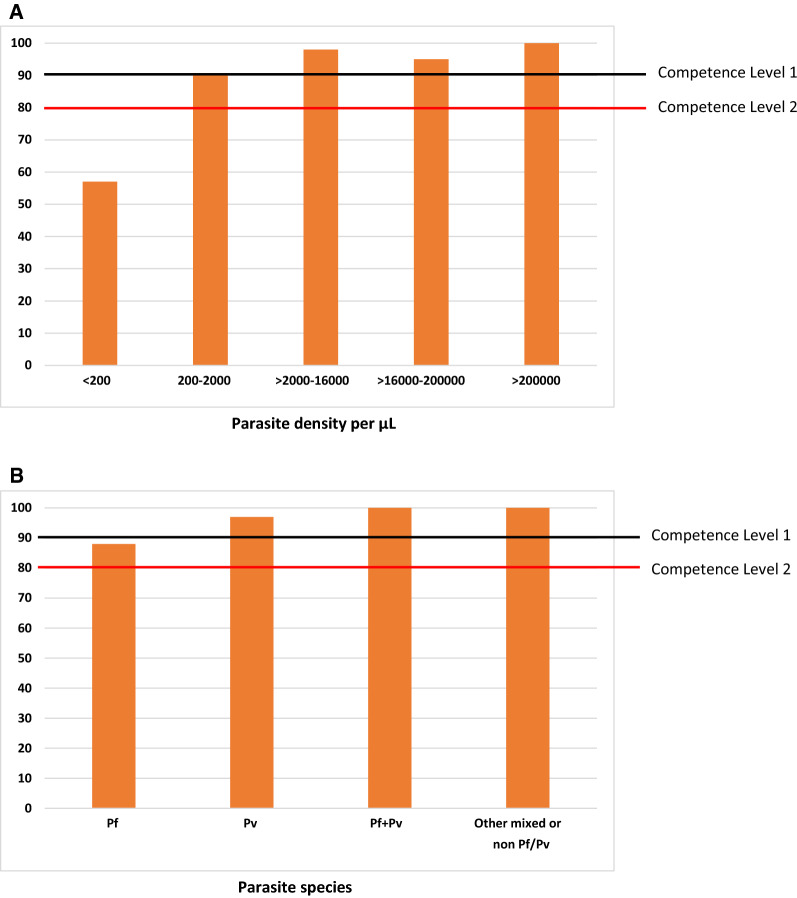
Sensitivity (%) of the EasyScan Go stratified by—**A** parasite density and **B** parasite species. Pf = Plasmodium falciparum; Pv = Plasmodium vivax

Compared to reference microscopy, the diagnostic sensitivity of the EasyScan Go device was 91.1% (95% CI 88.9–92.7) and specificity was 75.6% (95% CI 73.1–78.0) (Table 1). Sensitivity of the digital device was considered stratified into five parasite density groups: < 200, 200–2000, > 2000–16,000, > 16,000–200,000, > 200,000 p/µL. Sensitivity varied according to parasite density. It was 57.1% (n = 102, 95% CI 46.7–67.1) at < 200 p/µL, 90.2% (n = 181, 95% CI 84.7–94.2) at 200–2000 p/µL, 97.5% (n = 330, 95% CI 95.1–98.9) at > 2000–16,000 p/µL, 94.6% (n = 327, 95% CI 91.5–96.8) at > 16,000–200,000 p/µL, and 100% (n = 29, 95% CI 88.1–100.0) at > 200,000 p/µL (Fig. 2A). Diagnostic sensitivity for *P. falciparum*, *P. vivax,* and mixed *P. falciparum* and *P. vivax* infections were 87.9% (n = 624, 95% CI 85.0–90.4), 96.5% (n = 327, 95% CI 93.7–98.2), and 100% (n = 8, 95% CI 63.1–100.0), respectively (Fig. 2B).

**Table 1:** EasyScan Go diagnostic performance using microscopy as the reference standard

Parasite Detection	Slides, n (Pos, Neg)	Sensitivity, % (95% CI)	Specificity, % (95% CI)
Overall	2152* (929, 1223)	91.1 (88.9–92.7)	75.6 (73.1–78.0)
First tier sitesφ	1464 (585, 879)	89.1 (86.2–91.5)	85.1 (82.6–87.4)
Second tier sitesφ	688 (344, 344)	94.2 (91.2–96.4)	51.5 (46.0–56.8)

^*^EasyScan Go reading missing, n = 98 due to invalid results

Based on EQC reports of smear and stain quality, sites were assessed according to relative percentages of good vs lower quality slides (“first tier sites” vs.“second tier sites”)

### Species identification by the EasyScan Go

Among those positive samples that were labelled as positive, parasite species was identified accurately in 93% (499/537) of *P. falciparum* samples and in 92% (281/307) of *P. vivax* samples (counting a ‘mixed *P. falciparum* + *P. vivax*’ label as correct in either case; counting a ‘mixed’ label as incorrect gives 86% and 82% accuracies, respectively). For *P. falciparum* and *P. vivax* species identification, kappa coefficients were 0.76 (95% CI 0.69–0.83) and 0.73 (95% CI 0.66–0.80); Table 2.

**Table 2:** Parasite species identification by EasyScan Go

	All sites		First tier sites*		Second tier sites*	
Parasite Species	Slides, n	Kappa value, (95% CI)	Slides, n	Kappa value, (95% CI)	Slides, n	Kappa value, (95% CI)
*P. falciparum*	537	0.76 (0.69–0.83)	358	0.77 (0.68–0.85)	179	0.74 (0.63–0.85)
*P. vivax*	307	0.73 (0.66–0.80)	163	0.68 (0.60–0.77)	144	0.79 (0.68–0.90)

^*^Based on EQC reports of smear and stain quality, sites were assessed according to relative percentages of good vs lower quality slides (“first tier sites” vs.“second tier sites”)

### Parasite density estimation

**Fig. 3:**
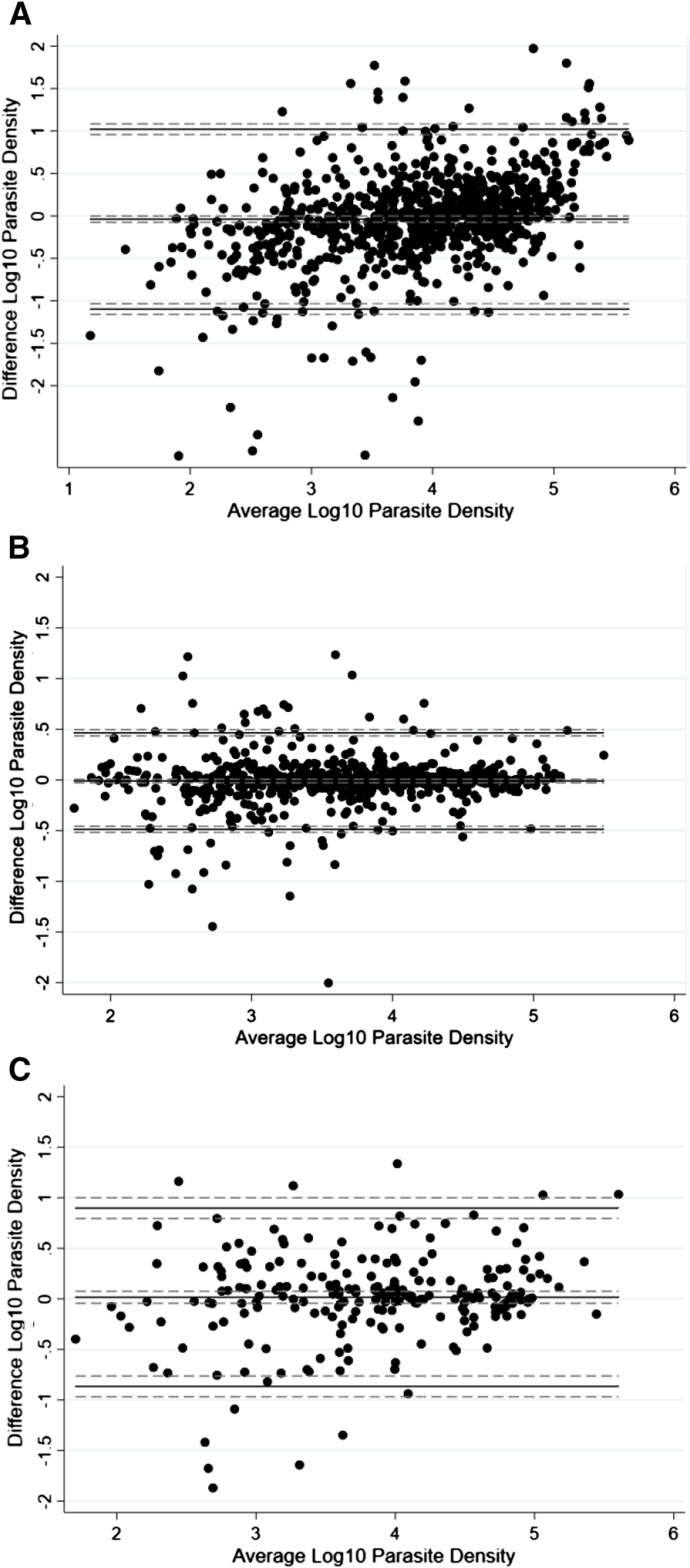
Bland–Altman plot for parasite density estimation: **A** Between microscopy and EasyScan Go (difference = microscopy count–EasyScan Go count); **B** Intra-device reliability–EasyScan Go 1st and 2nd reads; **C** Inter-device reliability–between two EasyScan Go devices

Parasite density estimates by the EasyScan Go were within ± 25% of the microscopic reference counts in 23% (196/845) of slides. A Bland–Altman plot for assessing agreement of parasite density estimations between microscopy and EasyScan Go, revealed a wide interval agreement ranging from − 1.098 to 1.021 in a logarithmic scale, corresponding to 0.08 to 10.50 in original scale with a mean difference of − 0.038 (n = 845, 95% CI − 0.075 to − 0.002) (Fig. 3A). Comparing parasite counts between the EasyScan Go 1st and 2nd readings (Intra-device reliability) showed the mean difference to be − 0.011 (n = 726, 95% CI − 0.029 to 0.007) and the limits of agreement − 0.487 to 0.465 on a logarithmic scale (0.33 to 2.92 in original scale) which corresponds to a maximum of roughly threefold over- or underestimation of parasite density (Fig. 3B). With respect to inter-device (field device vs another device in the reference laboratory in Bangkok) reliability assessment, the mean difference was − 0.016 (n = 222, 95% CI − 0.043 to 0.076) and the limits of agreement in a logarithmic scale − 0.865 to 0.897 corresponding to 0.14–7.89 in original scale (Fig. 3C). While there was low variance on average between microscopy and the EasyScan GO and also with respect to intra- or inter-device comparisons, the agreement intervals were large. These indicated that parasite density estimates from EasyScan GO were over- or underestimated by up to roughly tenfold as compared to microscopy. Even when comparing repeat reads of slides on the same or different devices, parasite density estimates varied by approximately 3- to eightfold, reflecting poor accuracy and precision in parasite density estimation overall.

### Slide quality sub-groups analysis

A major challenge for automated algorithms is distinguishing between parasites and artefacts, and low quality slides increase this difficulty. Thus differential performance may be expected based on slide quality. All prospective sites followed a standardized laboratory manual, SOPs, and stringent EQC procedures for malaria microscopy. However, in field settings there were variations in the staining and quality of smears. Based on EQC reports of smear and stain quality, sites were assessed according to relative percentages of good vs lower quality slides (“first tier sites” vs. “second tier sites”).

**Fig. 4:**
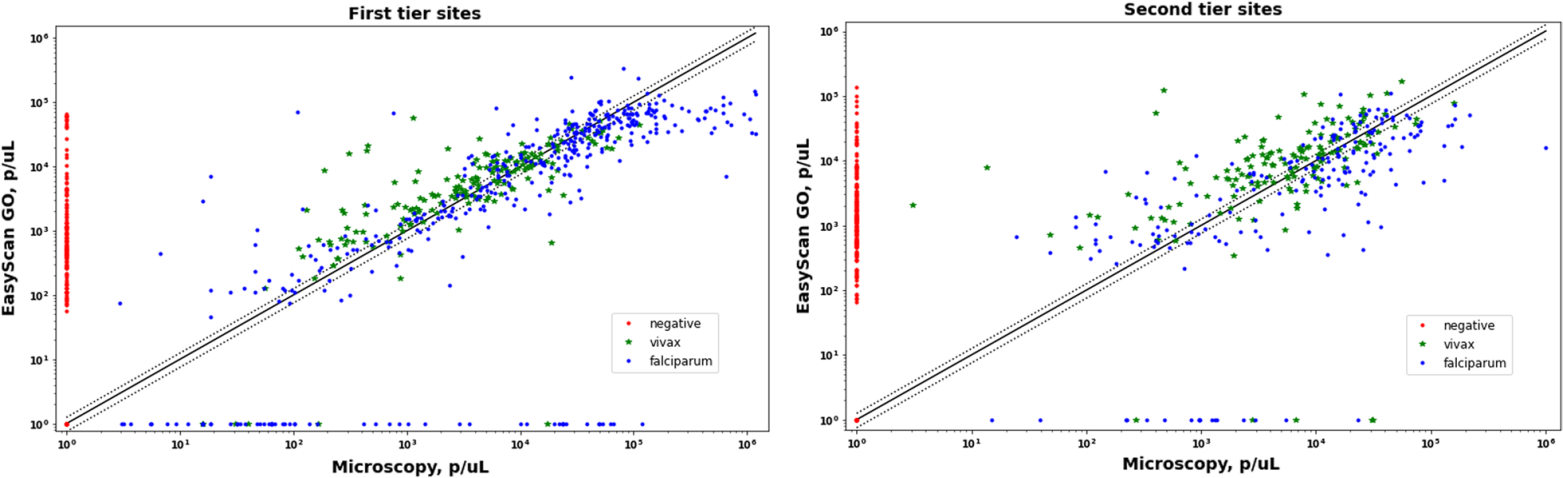
Estimated parasite density, EasyScan GO 1^st^ read vs Microscopy. Log–log plot. Left: First tier (quality) sites. Right: Second tier sites. False Positives are the red dots along the *y*-axis. False Negatives are the dots along the *x*-axis. The green dotted lines show ± 25% quantitation accuracy boundaries

There were 1478 slides from first tier sites (409 falciparum, 162 vivax, 8 mixed, 9 other, 890 negative) and 705 slides from second tier sites (198 falciparum, 156 vivax, 0 mixed, 1 other, 351 negative). Diagnostic sensitivity of the EasyScan Go was slightly lower on the first tier vs second tier sites, at 89.1% vs. 94.2% (5.1% difference (95% CI 2.6–7.4%), P = 0.0001), whereas specificity was much higher, at 85.1% vs 51.5% (33.6% difference (95% CI 29.4–37.7%), P < 0.0001). For *P. falciparum* species identification, kappa values remained similar (0.77 in first tier vs 0.74 in second tier), but for *P. vivax* species identification, kappa values were lower in the first tier sites (0.68 vs. 0.79). Quantitations were more accurate in first tier sites: 27% (141/521) of the parasite counts derived from the EasyScan Go were within ± 25% of the microscopic reference counts vs. 17% for second tier sites, a 10% difference (95% CI 4.2–15.4%, P = 0.0008). Figure 4 shows both first and second tier parasite density estimates.

## Discussion

This large field evaluation, conducted in varied geographical and epidemiological settings, represents the real-life performance of a fully automated digital microscope with thick-film-only malaria detection algorithms. Because none of the slides from the current evaluation were used in the training of the algorithms, the results given are true hold-out performance. An important aspect of this study was the inter-site slide preparation variability. Some sites (“first tier sites”) had overall higher quality slides than others (“second tier sites”) as assessed during EQC, enabling assessment of the effects of slide quality on algorithm performance.

On the 1478 slides from first tier sites, the device had overall parasite detection sensitivity 89%, specificity 85%, species identification accuracy 84% on positive-labelled slides (increasing to 90% if a ‘mixed’ label were counted as correct for *P. falciparum* or *P. vivax,*and vice versa), and 27% of quantitations within 25% error using expert microscopy as reference. Though this does not correspond to the criteria for expert microscopy, it represents reasonable performance, potentially useful in clinical settings.

On the 705 slides from second tier sites, overall sensitivity was 94%, but specificity dropped substantially to 52% in comparison with the first tier sites. Quantitation accuracy also dropped substantially with 17% of quantitations within the 25% error, whereas the corresponding figure for first tier sites was 27%. The lower specificity was in general due to abnormally high numbers of artefacts, which the algorithm labelled as parasites on poor quality slides. The algorithm assumes a certain noise floor (i.e. a certain FP object rate) based on assumptions about the expected numbers of parasite-like artefacts. If a slide presents many more parasite-like artefacts than expected, the noise floor is exceeded and an incorrect Positive slide disposition results (this can also lead to higher sensitivity, though sometimes for the wrong reason).

A somewhat strange finding pertaining to species identification of *P. vivax* samples was that second tier sites had a higher kappa value than first tier sites (0.79 vs. 0.68) due to fewer vivax samples being misclassified as falciparum. This might have been due indirectly to higher levels of late-stage-like artefacts in second tier sites—the algorithm determines species of a slide based largely on the ratio of ring-stage to late-stage suspected parasites, since late stages are so rare in falciparum samples. A few extra late-stage suspects caused by artefacts could push a sample to the vivax label, despite possibly high numbers of ring-stage suspects, increasing the odds that a low-quality vivax sample would be labelled vivax.

### Competency levels

This field evaluation demonstrated a parasite detection sensitivity of 89% and species identification accuracy of 84% on slides with acceptable quality when compared to expert microscopy. This corresponds to Level 2 criteria (80–90% for both sensitivity and species identification) as defined in the WHO-TDR Research Malaria Microscopy manual [21]. For comparison, the criteria for Level 1 (“Expert”) competency are sensitivity and species detection accuracy of > 90%. On the other hand, specificity was 85%, and only one-fourth of parasite counts by the automated digital device were within ± 25% of the microscopic reference counts, corresponding to a level 4 WHO-TDR criteria (Table 3) [21]. Diagnostic sensitivity varied with parasitaemia, falling to as low as 40% in individuals with parasitaemia less than 200 p/µL, but increasing to over 90% in those parasitaemia > 2000 p/µL. For parasite detection and species identification, the digital microscope showed good performance overall. Further software improvement is warranted to improve parasite density estimations, sensitivity at low parasite densities and diagnostic specificity.

**Table 3:** EasyScan Go ‘competence level’ as per the WHO-TDR Research Malaria Microscopy criteria [21]

Competence Level	Parasite detection (%)	Species identification (%)	Parasite count within 25% of true count (%)	False positive rate (%)
1	90–100	90–100	50–100	≤ 2.5
2	**80–89 [EasyScan Go]**	**80–89 [EasyScan Go]**	40–49	≤ 5
3	70–79	70–79	30–39	≤ 10
4	0–69	0–69	**0–29 [EasyScan Go]**	** > 10 [EasyScan Go]**

Parasite detection corresponds to sensitivity and false positive rate corresponds to 1–specificity. In the WHO Malaria Microscopy Quality Assurance Manual, sensitivity and specificity are combined as overall parasite detection accuracy [7]. Per this criterion, the performance of the EasyScan Go (parasite detection 82.3%) corresponds to Competence Level 2

### Effects of slide quality

The differential performance of the digital device depending on slide quality highlights the need for high quality slide preparation when performing automated microscopy, which was not achieved in roughly 30% of the slides from the sites, despite procedures and adequate training conducted by research groups. Considerable variations were observed in staining and blood film preparation across study sites. Expert microscopists were able to accurately process the “poor quality” slides in this study, consistent with findings in [16]. Thus, the issue of poor slide preparation is much more urgent for automated systems than for manual microscopy. This may partly explain the persistence of variable slide preparation issues: variable quality does not impact human microscopists to the same extent as it does automated systems wherein sensitivity to variable slide quality and staining is a central, difficult challenge. The current algorithm was trained on a diverse set of training slides from multiple sites. Adding training samples from more sites can slowly and expensively improve robustness; adjusting the ‘noise threshold’ for each site and rejecting results from slides flagged as being poor quality could be an interim measure (see below). However, new machine learning techniques that directly address slide variability and enable on-the-fly adaptation to new sites are likely to be better solutions, although it is difficult to predict if or when they can be achieved.

Standardization of blood film staining and preparation by means of automated staining might improve consistency in slide quality and thus aid automated systems. The automated staining technique with Giemsa stain, i.e. use of auto-spreading and auto-staining slides is challenging, expensive and not widely used. The EasyScan GO algorithms have a module that detects and flags potentially poor quality blood films. Such feedback to field clinicians might improve the device’s diagnostic accuracy by helping clinics address preparation issues affecting the device.

### Prospects

Automated malaria microscopy platforms based on computer vision and machine-learning have been in development for over the past two decades [8, 28]. Some computer vision malaria diagnostic systems, such as *Parasight* using fluorescent stain, have advanced to commercial platforms recently [29]. The EasyScan Go, now advanced to a production prototype and is built around low-cost Giemsa staining techniques following current microscopy standard practice. The use of Giemsa-stained blood films poses challenges for algorithm development, but has considerable advantages in terms of large scale deployment and ease of use in the field. The most advanced digital malaria technologies, including the EasyScan Go, have achieved a limit of detection (LOD) around 100–150 parasites per microliter, matching the performance of a field microscopist. Remaining challenges include improving LOD and parasite density estimation, better addressing ovale and malariae, and handling the issue of variable slide quality (Additional file 6: Table S2).

This study had several limitations. The algorithm does not perform parasite staging, nor does it distinguish sexual from asexual stages. Although the software tested in this study readily distinguishes falciparum from non-falciparum species, it does not differentiate between the non-falciparum species (*P. vivax*, *P. ovale* and *P. malariae*) since it processed only thick films. A thin film module has since been added to the EasyScan GO [17, 19]. However, it is unlikely that these factors would have affected the estimates of the performance parameters presented here. In addition, the study did not evaluate the performance of the EasyScan Go under routine field operation.

The results of this study provide considerable optimism for machine-learning-based algorithms to perform tasks that are currently dependent on highly trained technicians in malaria Quality Assurance (QA) programmes (7). This could be achieved through supplementing the 1st microscopy reading by the EasyScan Go 2nd reading, as well as cross-checking of blood slides in the context of a QC procedure in therapeutic efficacy studies. The QC for malaria microscopy is a tedious process that should be performed independently to verify microscopy results and identify systematic errors in anti-malarial drug efficacy assessments. Despite the importance of quality assurance of slide readings in the context of malaria research and routine surveillance, there are vast differences in operating procedures, including identifying and resolving discrepant results [30, 31]. The EasyScan Go device has the potential to perform as a QC tool in anti-malarial drug efficacy assessments. In the current evaluation, two versions of the algorithm (version1.15.17b and version1.11.6) were developed over the course of the trial. The results demonstrate that the accuracy of diagnostic specificity and parasite density estimation was improved with the newer version of the algorithm and suggest that it is possible to improve the performance further with additional software development work. For example, incorporating a dynamic update to noise floor parameters, based on a small batch of slides from a new site, might improve algorithm generalizability. Additionally, automated image recognition is a rapidly developing field and applying new techniques to this problem may improve performance. Such changes can be done without changes in the hardware or device. Enhanced performance in estimating parasite density would open the possibility of using the device for parasite clearance rate estimations, which are also labour-intensive.

## Conclusions

The digital malaria microscopy device EasyScan Go has the potential to facilitate cross-checking of blood slides as part of quality assurance. Further software improvement is required to improve parasite density estimations and sensitivity at low parasite densities. High quality of smears and staining is paramount to allow machine-learning-based image analysis to perform adequately. The aforementioned technological barriers need to be overcome prior to implementing the EasyScan Go as a diagnostic tool in malaria research settings.

## Supplementary Information

**Additional file 1.** STARD-2015-Checklist.

**Additional file 2.** Parasite Density Estimation.

**Additional file 3.** EasyScan Go.

**Additional file 4.** Thick Film EasyScan Go Output.

**Additional file 5.** Study Enrolment.

**Additional file 6.** Site Specific Results.

## Data Availability

The datasets used for analysis are available through the WWARN data-sharing platform (https://www.wwarn.org/working-together/sharing-accessing-data/accessing-data).

## Abbreviations

RDT: Rapid diagnostic test
HPF: High power field
WBC: White blood cell
FP: False positive
FN: False negative
SOP: Standard operating procedure
STARD: Standards for Reporting Diagnostic accuracy studies
EQC: External quality control
LOD: Limit of detection
WHO: World Health Organization
WWARN: WorldWide Antimalarial Resistance Network
