# Supplementary material for: Field evaluation of the diagnostic performance of EasyScan GO: a digital malaria microscopy device based on machine-learning
Source: Malar J. 2022 Apr 12;21:122. doi: 10.1186/s12936-022-04146-1 (PMC9004086; doi:10.1186/s12936-022-04146-1)

Field Evaluation of the Diagnostic Performance of EasyScan GO – A Digital Malaria Microscopy Device Based On Machine-Learning

Debashish Das[1,2,3,4], Ranitha Vongpromek[1,2,5], Thanawat Assawariyathipat[1,2,5], Ketsanee Srinamon[5], Kalynn Kennon[1,2,3], Kasia Stepniewska[1,2,3], Aniruddha Ghose[6], Abdullah Abu Sayeed[6], M Abul Faiz[7], Rebeca Linhares Abreu Netto[8], Andre Siqueira[9], Serge R. Yerbanga[10], Jean Bosco Ouédraogo[10], James J Callery[5], Thomas J Peto[3,5], Rupam Tripura[3,5], Felix Koukouikila-Koussounda[11], Francine Ntoumi[11], John Michael Ong'echa[12], Bernhards Ogutu[12], Prakash Ghimire[13], Jutta Marfurt[14], Benedikt Ley[14], Amadou Seck[15], Magatte Ndiaye[15], Bhavani Moodley[16], Lisa Ming Sun[16], Laypaw Archasuksan[17], Stephane Proux[17], Sam L Nsobya[18,19], Philip J. Rosenthal[20], Matthew P. Horning[21], Shawn K McGuire[21], Courosh Mehanian[21], Stephen Burkot[21], Charles B. Delahunt[21,22], Christine Bachman[21], Ric N. Price[3,5,14], Arjen Dondorp[3,5], François Chappuis[23], Philippe J Guérin[1,2,3], Mehul Dhorda[1,2,3,5]*

Additional File 2. Parasite density estimation a) By HPF method b) By WBC method

EasyScan GO Evaluation

WORKING PROTOCOL - MICROSCOPY BY HPF METHOD

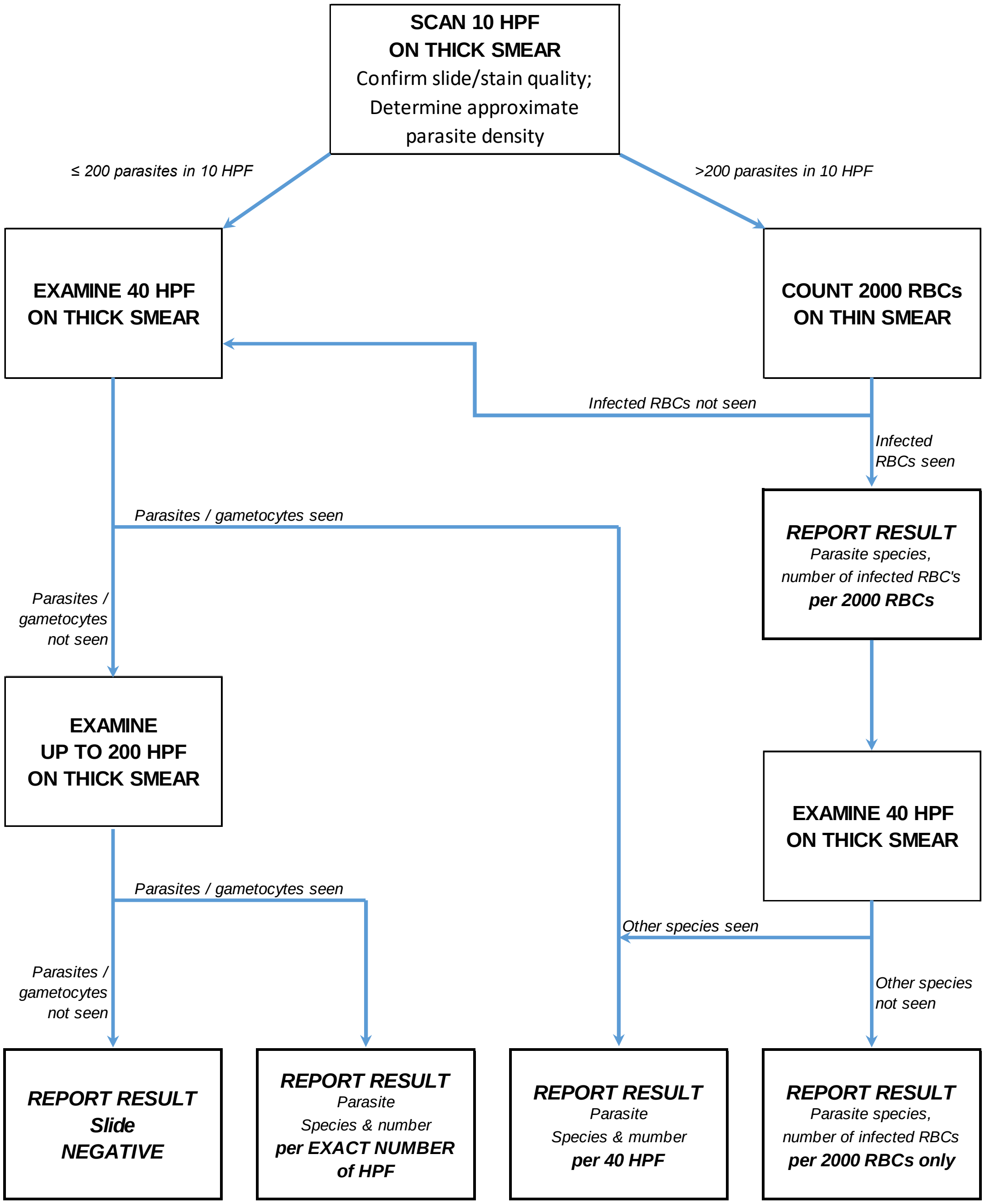

EasyScan GO Evaluation

WORKING PROTOCOL - MICROSCOPY BY WBC METHOD

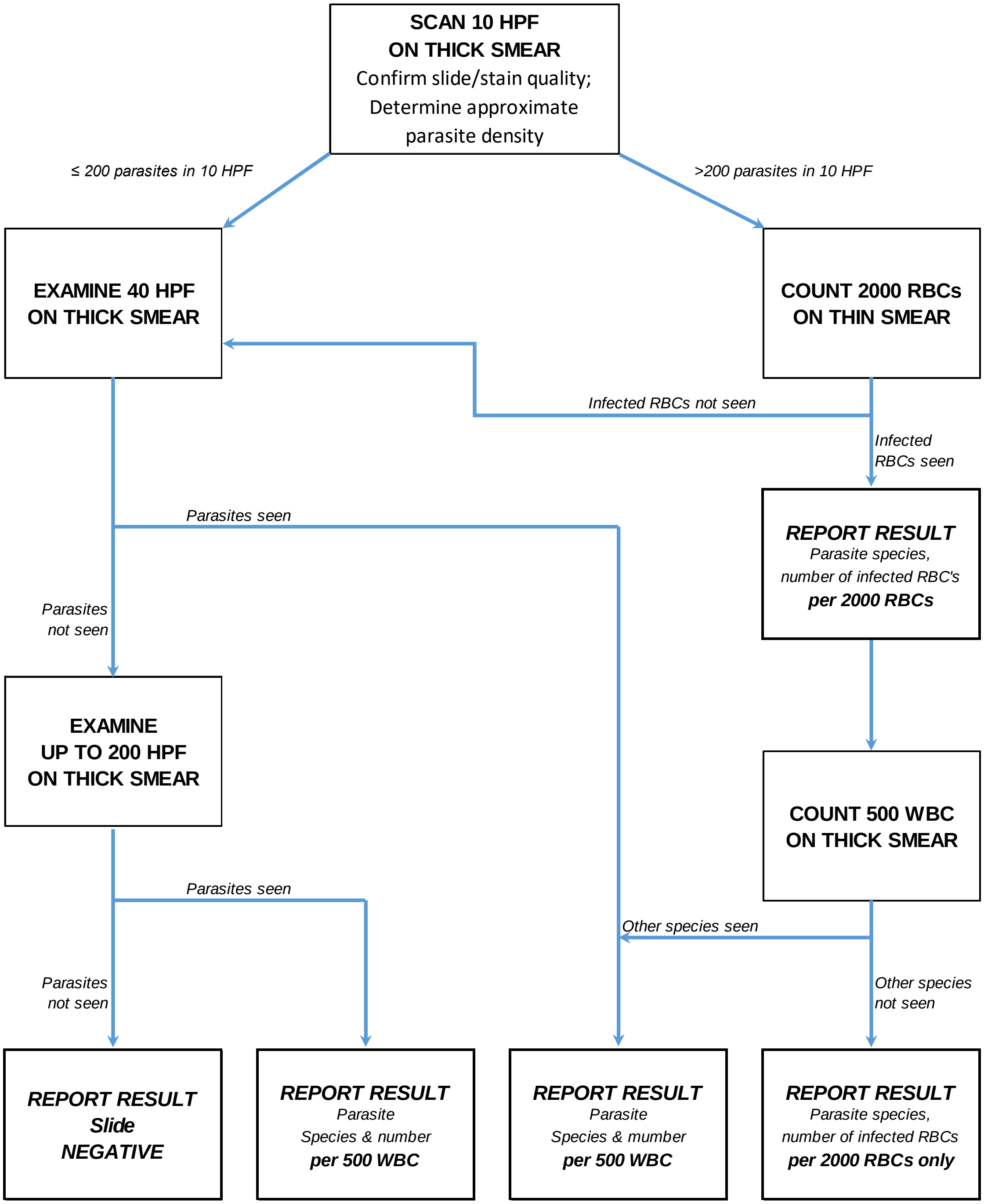

Supplement: Supplementary file 2 — Additional file 2. Parasite Density Estimation. [file 12936_2022_4146_MOESM2_ESM.pdf]
