# Supplementary material for: Field evaluation of the diagnostic performance of EasyScan GO: a digital malaria microscopy device based on machine-learning
Source: Malar J. 2022 Apr 12;21:122. doi: 10.1186/s12936-022-04146-1 (PMC9004086; doi:10.1186/s12936-022-04146-1)
Supplement: Supplementary file 3 — Additional file 3. EasyScan Go. [file 12936_2022_4146_MOESM3_ESM.pdf]

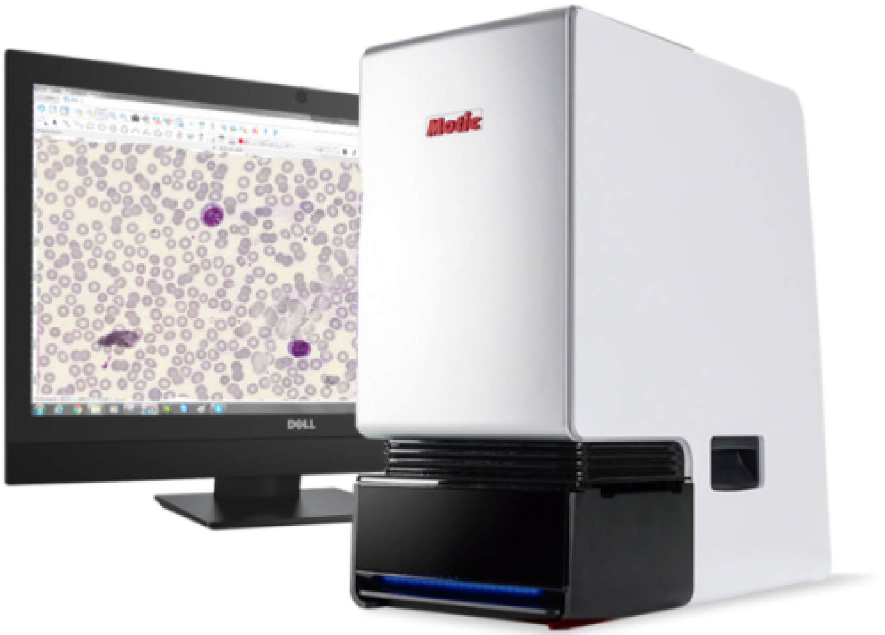

Additional Fig 3: EasyScan Go – a digital malaria microscope

(Source: MOTIC® Digital Pathology and Intellectual Ventures' Global Good Fund)
