# Supplementary figures and images for: Field evaluation of the diagnostic performance of EasyScan GO: a digital malaria microscopy device based on machine-learning
Source: Malar J. 2022 Apr 12;21:122. doi: 10.1186/s12936-022-04146-1 (PMC9004086; doi:10.1186/s12936-022-04146-1)

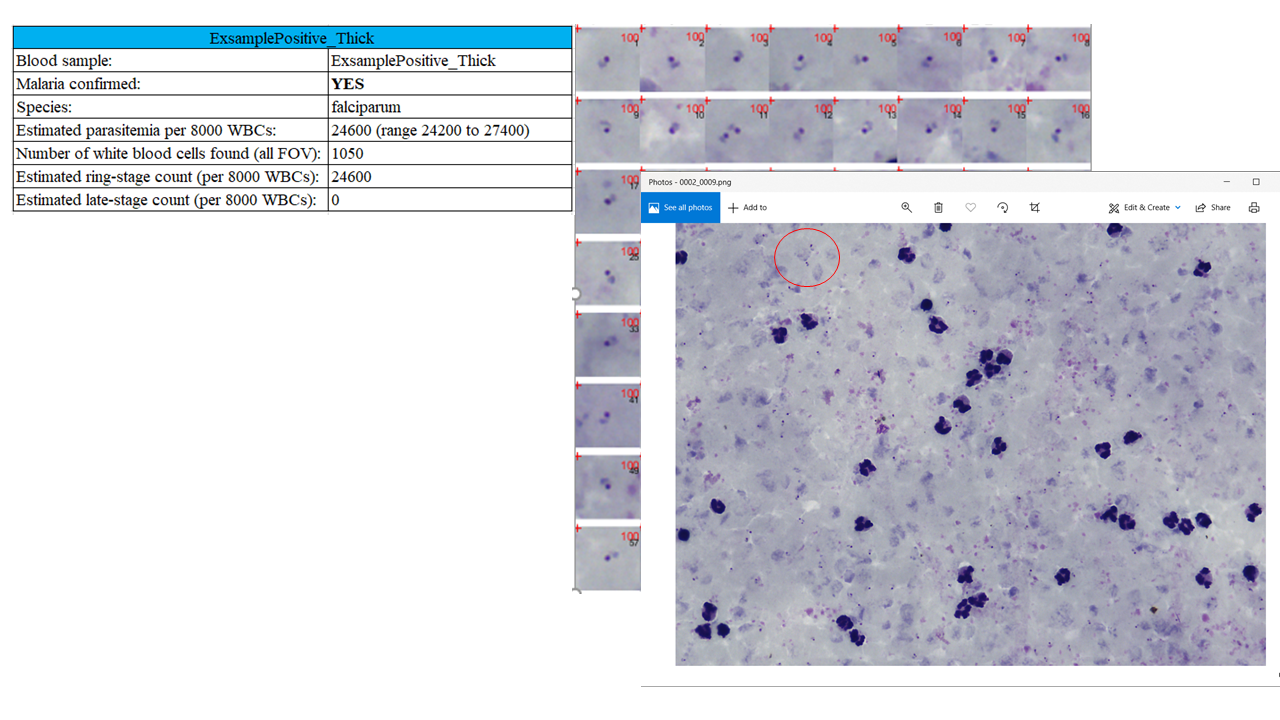

Supplement: Supplementary file 4 — Additional file 4. Thick Film EasyScan Go Output. [file 12936_2022_4146_MOESM4_ESM.tif]
